# Abrupt onset of suture granuloma 27 years after hemithyroidectomy

**DOI:** 10.1093/jscr/rjad284

**Published:** 2023-06-05

**Authors:** Ryan Alghanemi, Thomas Steinmüller

**Affiliations:** Department of Surgery, KMG Kliniken Luckenwalde, Luckenwalde, Germany; Department of Surgery, DRK Kliniken Berlin - Westend, Berlin, Germany

## Abstract

Suture granuloma is a rare complication after thyroidectomy and usually manifests as a chronic inflammation mimicking cancer or even tuberculous lymphadenitis and can be expected within the first 2 postoperative years. We report the case of a 53-year-old woman who presented, 27 years after her first hemithyroidectomy, with a sudden onset of a growing lump on the same site. Neck magnetic resonance imaging revealed a fast-growing tumor suggestive of a cancerous lesion. An excisional biopsy revealed only acute inflammation with pus formation. During surgery, we excised 20 thickly ligated sutures from the neck. These sutures were suggested to have caused the suture granulomas.

## INTRODUCTION

Suture granuloma is a localized inflammatory reaction responding to retained suture material, usually nonabsorbable material such as silk. However, with the introduction of resorbable sutures, developing suture-related complications has become a rare event, particularly after thyroidectomy [1]. Suture granulomas can develop years after thyroidectomy, manifesting signs of acute infection such as redness, swelling, tenderness or mimicking thyroid cancer, as observed in this case [2].

## CASE REPORT

**Figure 1:**
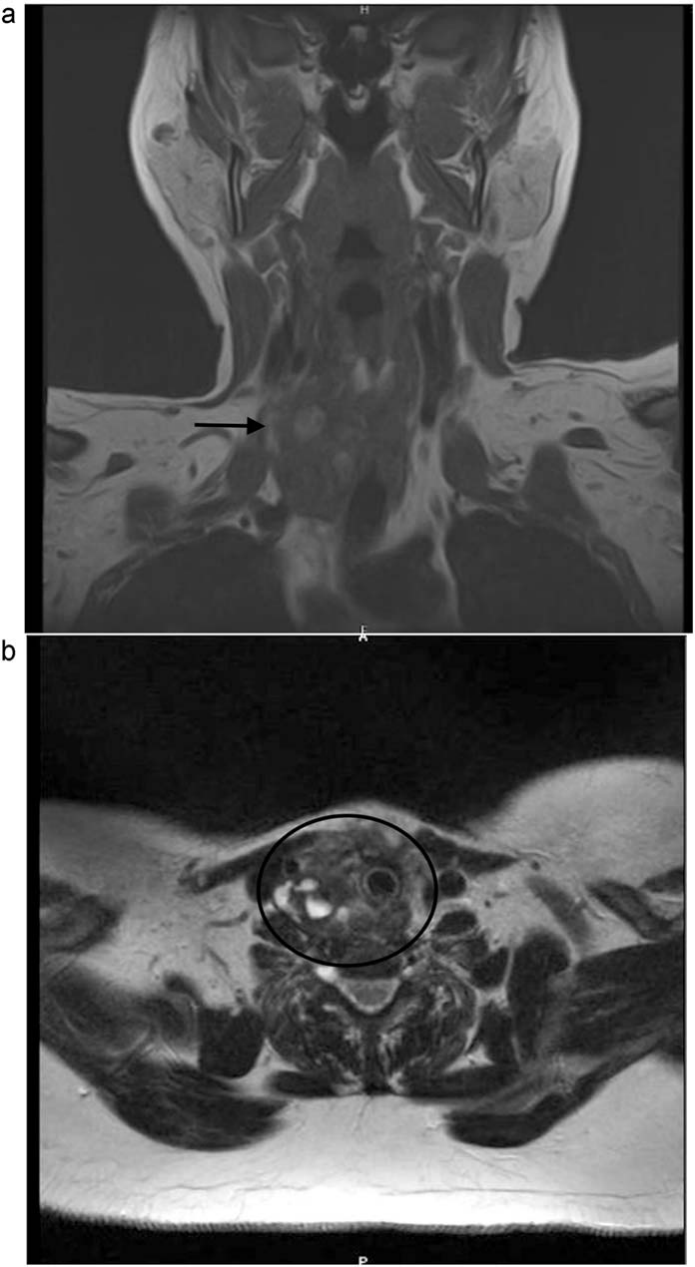
(**a**, **b**) Neck MRI, showing a mass 3 × 3.2 × 4.8 cm at the right thyroidectomy bed.

**Figure 2:**
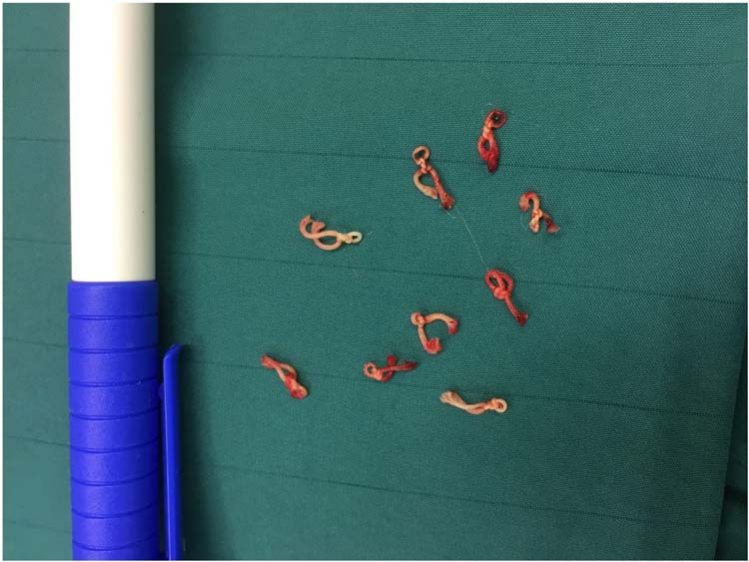
Specimen of the removed thick ligation sutures.

A 53-year-old Caucasian German woman with a medical history of hypertension and depression presented with a rapidly growing neck swelling on the right side, accompanied by a degree of induration. She had undergone right hemithyroidectomy 27 years ago for nodular goiter, resulting in right-sided recurrent laryngeal nerve palsy. However, 15 years prior to her presentation at our hospital, she underwent a left-sided hemithyroidectomy without complications. All thyroid function tests showed results within the normal ranges. Magnetic resonance imaging revealed a suspicious mass measuring 3 × 3.2 × 4.8 cm on the right side of the thyroid gland (Fig. 1). Concerns regarding the possibility of thyroid remnant and potentials for thyroid malignancy have been raised. The patient underwent surgical excision of the inflamed tumor from the neck musculature. The tumor was successfully extracted along with a staggering 20 thick ligation sutures, and the mini-abscesses were also managed (Fig. 2). During surgery, a tiny, benign thyroid remnant was found. Histological examination revealed a chronic fibrosing suture granuloma, with some areas showing focal active inflammation and foreign body reaction. Postoperative follow-ups showed marked improvement in the patient’s symptoms, without tumor recurrence.

## DISCUSSION

Regarding thyroidectomy, similar to other surgical procedures, possible complications such as suture granuloma could occur. Suture granuloma is a rare complication of surgery and defined as a localized inflammatory reaction in response to retained suture material, which is usually nonabsorbable. It is usually a slow inflammatory process, taking up to approximately 2 years to develop. However, variations of this rule have been reported, such as in our case [3]. Initially, the tissue might respond to trauma inflicted by the passage of surgical needle, resulting in an inflammatory reaction. Subsequently, the suture material could trigger a specific inflammatory response leading to granuloma formation [4]. This lesion might mimic tuberculous lymphadenitis [5] or a malignant tumor [2]. Moreover, clinical and imaging findings could be misleading. Although fine-needle aspiration biopsy is recommended [3], surgical excision remains the standard management for excluding malignancy and removing tumor. The material of the nonabsorbable sutures plays a key role in developing this complication [1]. In our case, 20 thick ligation sutures were excised, wherein signs of mini-abscesses were found and managed.

## CONCLUSIONS

Suture granulomas can develop years after thyroidectomy, presenting with signs of acute infection and mimicking thyroid cancer. Excisional biopsy is the gold standard for definitively excluding cancer and should be considered in cases of suspected suture granuloma. Early diagnosis and treatment of suture granuloma could help prevent further complications such as infection or abscess formation.

## CONFLICT OF INTEREST STATEMENT

None declared.

## FUNDING

None.
